# On the Origin of Indonesian Cattle

**DOI:** 10.1371/journal.pone.0005490

**Published:** 2009-05-13

**Authors:** Kusdiantoro Mohamad, Mia Olsson, Helena T. A. van Tol, Sofia Mikko, Bart H. Vlamings, Göran Andersson, Heriberto Rodríguez-Martínez, Bambang Purwantara, Robert W. Paling, Ben Colenbrander, Johannes A. Lenstra

**Affiliations:** 1 Faculty of Veterinary Medicine, Bogor Agricultural University, Bogor, Indonesia; 2 Faculty of Veterinary Medicine, Utrecht University, Utrecht, The Netherlands; 3 Department of Animal Breeding and Genetics, Swedish University of Agricultural Sciences (SLU), Uppsala, Sweden; 4 Department of Microbiology and Medical Biochemistry, Uppsala University, Uppsala, Sweden; 5 Division of Reproduction, Swedish University of Agricultural Sciences (SLU), Uppsala, Sweden; American Museum of Natural History, United States of America

## Abstract

**Background:**

Two bovine species contribute to the Indonesian livestock, zebu (*Bos indicus*) and banteng (*Bos javanicus*), respectively. Although male hybrid offspring of these species is not fertile, Indonesian cattle breeds are supposed to be of mixed species origin. However, this has not been documented and is so far only supported by preliminary molecular analysis.

**Methods and Findings:**

Analysis of mitochondrial, Y-chromosomal and microsatellite DNA showed a banteng introgression of 10–16% in Indonesian zebu breeds. East-Javanese Madura and Galekan cattle have higher levels of autosomal banteng introgression (20–30%) and combine a zebu paternal lineage with a predominant (Madura) or even complete (Galekan) maternal banteng origin. Two Madura bulls carried taurine Y-chromosomal haplotypes, presumably of French Limousin origin. In contrast, we did not find evidence for zebu introgression in five populations of the Bali cattle, a domestic form of the banteng.

**Conclusions:**

Because of their unique species composition Indonesian cattle represent a valuable genetic resource, which potentially may also be exploited in other tropical regions.

## Introduction

Several bovine species have contributed worldwide to cattle livestock [1]. Most domestic cattle belong to the species *Bos taurus* or *Bos indicus* (zebu), which both descend from the wild aurochs (*Bos primigenius*). Domestic yak (*Bos grunniens*) is kept in and around Tibet, the gayal (*Bos frontalis*) of Eastern India is derived from the gaur (*Bos gaurus*), while the Indonesian Bali cattle is a domestic form of the banteng (*Bos javanicus*). Despite their obvious role as livestock during our cultural development, the history of domestic cattle has been poorly documented. For the past 15 years, DNA analysis has allowed a phylogenetic reconstruction of the earliest events during domestication [2], [3]. For instance, analysis of mitochondrial DNA established a taurine maternal origin of zebu breeds outside Asia [4], [5]. Indonesian cattle breeds are supposed to be derived from zebu as well as from banteng [6].

Domestic Bali cattle is kept on Bali, East Java and on isolated regions on Sumatera and Sulawesi. It offers the advantage of a high resistance against most diseases, a remarkable ability to grow on low-quality fodder and a high fertility [7]. On the other hand, Bali cattle cannot be reared very well together with sheep because of their susceptibility to malignant catarrhal, while juvenile mortality is relatively high. A deer-like temperament makes them most suitable for intensive village-based management for plowing rice paddy fields [8], but their hoofs are too soft for draught on paved roads. Meat from young animals has a reputation of being exceptionally tender.

Crosses of banteng and zebu produce viable offspring, but male hybrids are not fertile [1]. However, the mixed banteng zebu species origin is not supported by breeding records, while only sporadic molecular data are available [9], [10], [11], [12], [13], [14]. Via an analysis of the maternal, paternal and autosomal species origin of five zebu breeds and five populations of Bali cattle, we show here that the species composition of Indonesian zebu breeds is unique and varies from mainly zebu to completely banteng. This information is of direct relevance for the genetic management and conservation of Indonesian cattle breeds.

## Materials and Methods

### Samples and DNA isolation

All animals were handled by veterinarians from the Faculty of Veterinary Medicine, Bogor Agricultural University in strict accordance with good animal practice following the guidelines of the Institutional Animal Care and Use Committee of Utrecht University. Blood and skin tissue samples from Bali cattle and banteng and blood samples from zebu breeds were collected on different locations (table 1). Blood and skin tissue samples from 8 bantengs were obtained from Ragunan Zoo, Jakarta. DNA was isolated by using standard SDS/proteinase K extraction [15] or the Qiagen blood and tissue extraction kit (Qiagen, Valencia, USA).

**Table 1:** Genetic constitution of Indonesian and Indian cattle breeds.

Breed/population	sampling site		samples		microsatellites			
	country/isle	location	males	females	genotypings	*H*_exp_	*H*_obs_	% banteng*
Ongole	India				32	0.64	0.67	
Nellore	Brasil				27	0.63	0.62	
Tharparkar	India				35	0.6	0.63	
Red Sindi	India				35	0.7	0.69	
Sahiwal	India				10	0.58	0.56	
Desi	India				24	0.65	0.66	
Hariana	India				10	0.68	0.67	
Aceh	North Sumatera	Langsa	8	3	11	0.68	0.68	10.8
Pesisir	West Sumatera	Painan	17	8	24	0.65	0.6	15.7
Filial Ongole	South Sumatera	Palembang		5	5	0.78	0.72	16.3
Galekan	East Java	Trenggalek	36	10	16	0.73	0.69	22.0
Madura	Madura	Bangkalan	18	23	28	0.75	0.65	31.3
Bali cattle	West Sumatera	Sijunjung	15	23	20	0.67	0.59	
Bali cattle	South Sumatera	Palembang	24	9	31	0.62	0.58	
Bali cattle	South–West Sumatera	Bengkulu	5	3	5	0.7	0.58	
Bali cattle	Sulawesi	Kendari, Kanowe Selatan	9	21	29	0.64	0.56	
Bali cattle	Bali	Denpasar, Tabanan	25	33	31	0.61	0.58	
Banteng	Java	Ragunan zoo, Jakarta	6	2	8	0.37	0.39	

* from the q value (Pritchard *et al.*, 2000) after analysis with Indian zebus and Bali cattle as predefined clusters.

### PCR-RLFP and sequencing

PCR-RLFP on a mitochondrial cytochrome *b* gene segment was carried out as described previously [16] with separate digestions by *Xba*I and *Taq*I ,indicating a zebu and banteng origin, respectively. In all cases, the separate digestions of mitochondrial DNA agreed and ruled out mistypings by genetic polymorphisms or failure of the digestions. PCR-RFLP of a Y-chromosomal *SRY* gene segment, in which a *Bfa*I site indicates a banteng origin (table 2), was performed as described [16]. The absence of the *Bfa*I site in undigested samples was confirmed by sequencing the same *SRY* fragment, which also indicated second banteng-specific mutation and differentiated between zebu and taurine origin [17]. For two samples a taurine origin was confirmed by sequencing intron 10 from the *ZFY* gene segment [17], [18]. For these samples, an indel in the same intron [18] as well as genotyping by K-Bioscience (Hoddesdon, UK) of single-nucleotide polymorphisms (SNPs) in exon 11 of *ZFY* and in *UTY* [18], [19] differentiated between the taurine Y1 and Y2 haplotypes. Table 2 summarizes the Y-chromosomal species variation and haplotype variation.

**Table 2:** Y-chromomal sequence variation diagnostic for indicine and Y1 and Y2 taurine haplotypes.

Gene	*SRY*			*ZFY*			*UTY*
Genbank entry	DQ336526			DQ336536		DQ336546	AY936543
Position	2059*	2100	2144	614	697–698	71	423
Taurine Y1	A	C	T	C	deletion	G	G
Taurine Y2	A	C	T	C	TG	T	T
Zebu	A	T	T	T	TG	T	T
Banteng	G	C	C	T	TG	T	T

* Corresponding to the *Bfa*I site in banteng.

Database entries and the numbering refer to sequences from taurine cattle.

### Microsatellite genotyping

Microsatellite analysis of the loci *INRA63*, *INRA5*, *ETH225*, *ILSTS5*, *HEL1*, *INRA35*, *ETH152*, *ETH10*, *CSSM66*, *ETH3*, *BM2113*, *BM1824*, *HEL13*, *BM1818*, *ILSTS6* and *CSRM60* was carried out using 10 ng of genomic DNA, 2 µM of M13 tailed forward primer, 10 µM of reverse primer, 10 µM of M13 oligonucleotide coupled to a fluorescent dye, *Taq* DNA polymerase and a standard PCR protocol. Fragments were separated on an ABI 3100 apparatus (Applied Biosystems, Foster City, CA). Allele size lengths have been standardized via comparison with a common reference sample. Data from Indian zebu breeds are from ref. [20].

### Data analysis

Checking of microsatellite data and calculation of expected heterozygosities was performed using the Excel-based microsatellite toolkit (http://animalgenomics.ucd.ie/sdepark/ms-toolkit/). Nei standard genetic distances were calculated using the program Microsat (http://hpgl.stanford.edu/projects/microsat/). NeighborNet graphs were constructed by the program Splitstree (http://www.splitstree.org/, [21]). Model-based clustering was carried out using the program Structure (http://pritch.bsd.uchicago.edu/software.html , [22], assuming admixture and correlated allele frequencies. Reproducible clustering was obtained after 30,000 burnin steps and 40,000 simulations. Clusters were either inferred or predefined as Indian zebu and Bali cattle, respectively. Results were displayed by the program Distruct (http://rosenberglab.bioinformatics.med.umich.edu/distruct.html, [23]).

## Results

**Figure 1:**
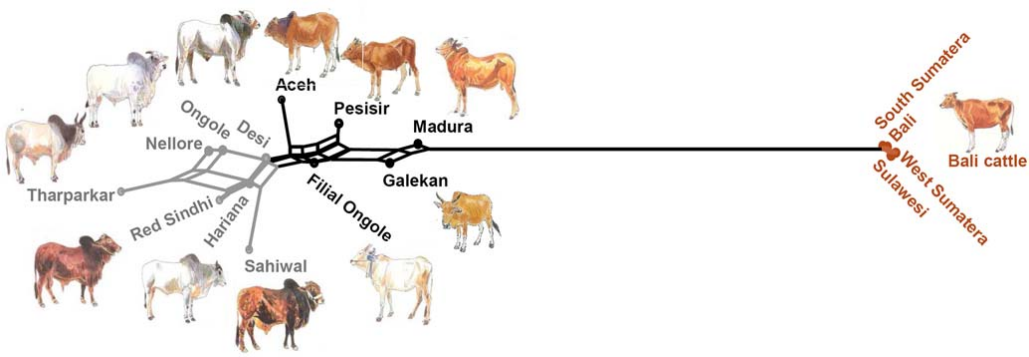
Locations of sampling and genetic constitution of Indonesian cattle populations. The species origin of the Y-chromosomes (Y), mitochondrial DNA (mt) and autosomal microsatellite alleles (μst) is represented by brown and gray shading of the indicated circle segments.

**Figure 2:**
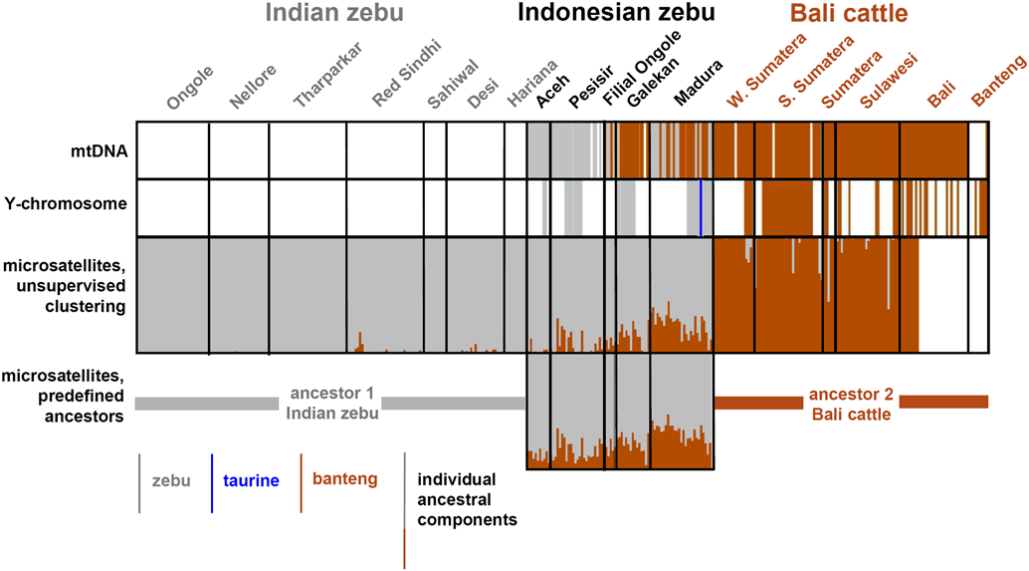
Genomic components of Indonesian cattle animals. Animals are represented as vertical lines, the color of which indicates zebu of banteng mitochondrial DNA (top panel), zebu, taurine or banteng Y-chromosomal DNA (second panel, only for males), the proportion of the individual genomes derived from the two clusters identified by unsupervised model-based clustering (third panel) or from the proportions derived from two predefined clusters (Indian zebu and Bali cattle, bottom panel). Each animal is plotted in the respective panels at the same position. A white line indicates absence of data.

As indicated by specific PCR-RFLP assays and sequencing, the sampled Aceh and Pesisir zebus have zebu mitochondrial DNA, while maternal lineages from both species are represented in Filial Ongole cattle (figures 1 and 2). In two earlier studies, banteng mtDNA has been found in 20 out of 26 [9] or six our of seven [10] Filial Ongole animals. We found banteng mtDNA also in 56% and 94% of the East-Javanese Madura and Galekan zebu samples, respectively. However, the maternal origin of Bali cattle from five different locations on three isles is almost exclusively banteng with a zebu origin found for only 1 out of 125 sampled animals. This is in contrast to the mixed maternal origin of Bali cattle from Malaysia [13], but agrees with the results obtained for a feral population of Bali cattle [24].

Interestingly, Y-chromosomal typing as a probe of the paternal lineage does not completely parallel the mtDNA results (figure 1 and 2). All zebu bulls carry exclusively zebu Y-chromosomes. Only female Filial Ongole animals were sampled, but in another study [10] seven bulls from this breed were found to carry zebu Y-chromosomes. Zebu Y-chromosomes were also found in the East-Javanese Galekan and Madura breeds. However, for two Madura bulls the sequence of the *ZFY* and *SRY* gene segments (table 2) indicate a taurine origin, possibly resulting of experimental crosses with Danish Red and French Limousin bulls [8]. These two European breeds carry different Y-chromosomal haplotypes (Y1 and Y2, respectively, [19]). Different diagnostic SNPs (table 2) revealed that both Madura bulls with taurine Y-chromosomes carried the Y2 haplotype, compatible with a Limousin origin. The parental origin of Madura cattle may also depend on the sampling site, because we previously found banteng Y-chromosomes in two Madura bulls from a breeding station in Malang on Java [14]. With one exception in South Sumatera, all Bali cattle in our study descend from banteng bulls.

For estimation of the autosomal species composition, we genotyped 16 microsatellite markers from the panel of 30 microsatellites recommended by the FAO for diversity studies (http://lprdad.fao.org/cgi-bin/getblob.cgi?sid=-1,50006220) and compared the data with genotypes for the same markers from seven Indian zebu populations [20]. As indicated by the expected heterozygosity, genetic diversity of Indonesian cattle compares to that of Indian (table 1). In Bali cattle observed heterozygosity is clearly lower than the expected values, presumably because of inbreeding within local populations. However, clearly higher heterozygosity values were observed for the Indonesian Ongole, Madura and Galekan, while the lowest value found for eight wild bantengs probably indicates inbreeding in a zoo population.

**Figure 3:**
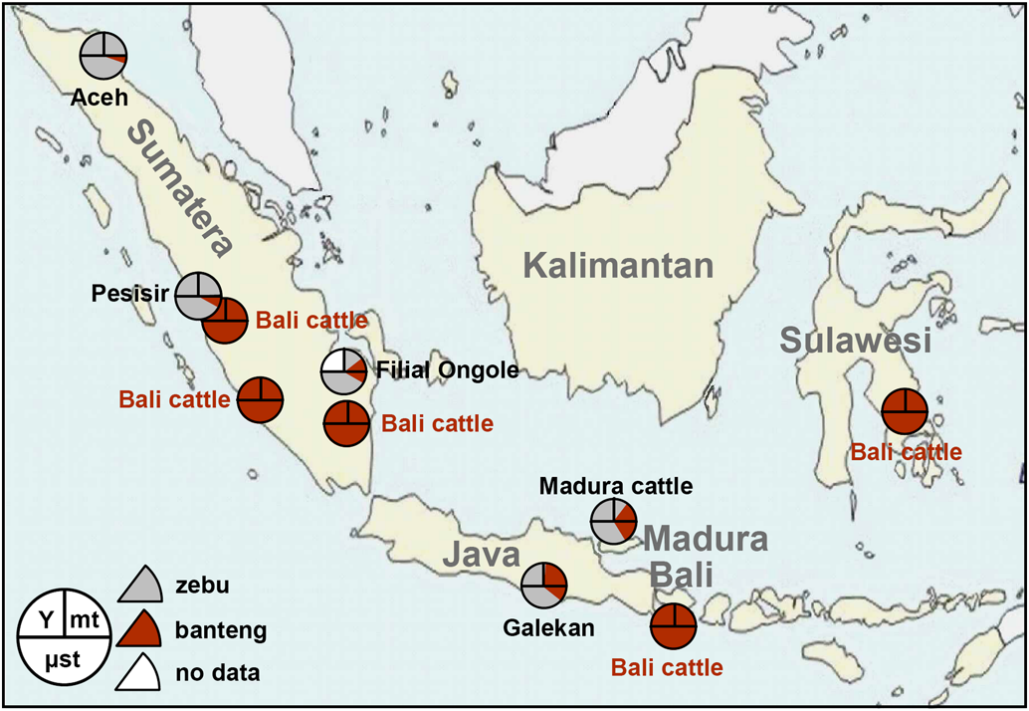
NeighborNet graph of genetic distances of Indonesian cattle populations. The animal pictures are from [8] or were drawn from photographs.

Allele distributions (not shown) of Indonesian and Indian breeds also matched well. However, for several markers additional alleles also present in Bali cattle were observed. Quantitative species components were estimated by two different methods (see [25]). First, Nei genetic distances were visualized in a Neighbor Network (figure 3), Indonesian Aceh, Pesisir and Filial Ongole are close to the Indian zebu breeds, but are intermediate between Indian zebu and Bali cattle. Interestingly, Madura and Galekan cattle, several of which carry banteng mitochondria, are more distant from the Indian zebu and closer to Bali cattle. The different Bali cattle populations appear to be identical. Adding genetic distances to the captive banteng population links this population to Bali cattle with a distance that probably corresponds to their inbreeding (not shown).

Second, unsupervised model-based clustering, i.e., without prior information on ancestral clusters, [22] identified a zebu and a Bali cattle cluster (figure 2). Assuming that Indian zebu and Bali cattle are the ancestral populations we then carried out supervised clustering in order to estimate the levels of introgression via the individual membership coefficients (figure 2, table 1). For Aceh, Pesisir and Filial Ongole banteng introgression is in the range of 11–16%, but this is clearly higher for Galekan (22%) and Madura cattle (31%).

## Discussion

Domestication of banteng probably took place around 3500 years BC [6], [8]. Bali cattle is currently the main representative of the domestic banteng, and it is kept on several Indonesian Isles. A cattle population in Pandaan on East Java is also supposed to originate from banteng (T. Susilawati, Malang, pers. comm.). There is no reliable dating of the entry of the more common cattle species, which in Indonesia was very likely predominantly of zebu origin ([26], [27]). Singalese immigrants may 1500 years ago have brought Indian cattle ([8]). Statues of a humped bull on Javanese Hindu temples evidence the presence of zebus in the 10^th^ century. Photographs of original Javanese cattle from the beginning of the 20^th^ century ([28], [29]) also show humped cattle and resembled Madura cattle. However, starting by the end of the 19^th^ century, imported Ongole zebu were more and more used for breeding on Java and other Indonesian isles, but not on the isles of Madura and Bali [6], [8], [28].

The predominance of zebu mitochondria in the Indonesian zebu breeds shows that not only zebu bulls but also zebu cows were imported. This is in contrast to the zebu populations in Africa and America, which emerged by systematic crossing of imported zebu bulls with taurine cattle [4], [5]. Banteng mitochondria in Indonesian Ongole populations as well as the autosomal microsatellite analysis indicate crossbreeding of zebu and local cattle with a banteng maternal origin.

The Eastern Java Galekan cattle are supposed to descend from original Java cattle (T. Susilawati, personal communication). DNA analysis indicates that it descends from banteng cows and zebu bulls. Madura has long tradition of well managed cattle husbandry [28], [30], [31]. The Madura breed predates the import of Ongole bulls and Madura bulls were used for crossing on East Java before the import of Ongole bulls [28]. DNA analysis of Madura cattle shows a combination of a zebu paternal lineage with a mixed zebu-banteng maternal origin. Experiments of upgrading the local cattle with Danish Red and Limousin taurine bulls, which have similar coat colors [8] were not pursued, but apparently left taurine Y-chromosomes in the Madura population. Although indiscriminate cross-breeding with exotic breeds is a major threat to the conservation of genetic resources, it is also evident that introgression of foreign material at such a low level in this case did not affect the identity of the breed.

Our data further indicate that Bali cattle on different locations in Indonesia has been kept separate from zebu, this in contrast to mixed zebu-banteng Bali cattle populations from Malaysia [13].

Evidently, the history and breeding of Indonesian cattle has resulted in a unique genetic resource that combines the general tolerance of zebu to tropical and dry climates with the adaptation of domestic banteng to Indonesian conditions and husbandry. Information about the history and species composition as reported here appears most essential for strategic choices regarding breed management and conservation. Furthermore, the adaptation of Indonesian cattle to different modes of management under tropical conditions may very well be exploited outside Indonesia, especially if the high-temperature zones expand because of current global climate trends [32].
