# Correction: On the Origin of Indonesian Cattle

**DOI:** 10.1371/annotation/e674f274-4378-47f1-97e1-f95a2d3bb59b

**Published:** 2009-05-19

**Authors:** Kusdiantoro Mohamad, Mia Olsson, Helena T. A. van Tol, Sofia Mikko, Bart H. Vlamings, Göran Andersson, Heriberto Rodríguez-Martínez, Bambang Purwantara, Robert W. Paling, Ben Colenbrander, Johannes A. Lenstra

The images for Figures 1 and 3 were incorrectly switched. The image that appears as Figure 1 should be Figure 3, and the image that appears as Figure 3 should be Figure 1. The figure legends appear in the correct order.

